# FAS/FASL Expression Profile as a Prognostic Marker in Squamous Cell Carcinoma of the Oral Cavity

**DOI:** 10.1371/journal.pone.0069024

**Published:** 2013-07-19

**Authors:** Paulo Bentes de Carvalho-Neto, Marcelo dos Santos, Marcos Brasilino de Carvalho, Ana Maria da Cunha Mercante, Viviane Priscila Pina dos Santos, Patrícia Severino, Eloiza Helena Tajara, Iuri Drumond Louro, Adriana Madeira Álvares da Silva-Conforti

**Affiliations:** 1 Serviço de Cirurgia de Cabeça e Pescoço, Hospital Heliópolis, São Paulo, São Paulo, Brazil; 2 Programa de Pós Graduação em Biotecnologia, Universidade Federal do Espírito Santo, Vitória, Espírito Santo, Brazil; 3 Laboratório de Biologia Molecular, Hospital Heliópolis, São Paulo, São Paulo, Brazil; 4 Departamento de Anatomia Patológica, Hospital Heliópolis, São Paulo, São Paulo, Brazil; 5 Centro de Pesquisa Experimental, Instituto Israelita de Ensino e Pesquisa Albert Einstein, São Paulo, São Paulo, Brazil; 6 Departamento de Biologia Molecular, Faculdade de Medicina, São José do Rio Preto, São Paulo, Brazil; Centro di Riferimento Oncologico, IRCCS National Cancer Institute, Italy

## Abstract

FAS/FASL altered expression may cause tumor protecting immunomodulation, with a direct impact on patient prognosis. FAS expression was studied in 60 squamous cell carcinomas of the oral cavity. FAS expression did not show a significant association with tumor histopathological characteristics, but was significantly associated with lymph node positivity. FAS expression was significantly associated with disease specific death and negative FAS expression was an independent risk factor, increasing risk 4 times when compared to positive expression. When FAS and FASL expression results were combined, we were able to define high, intermediate and low risk profiles. Disease-free and disease-specific survival were significantly correlated with FAS/FASL expression profiles. The high risk category was an independent marker for earlier disease relapse and disease-specific death, with approximately 4- and 6-fold increased risk, respectively, when compared to the low risk profile. Risk profiles based on FAS/FASL expression showed that high risk was significantly associated with increased disease relapse and death, as well as shorter disease-free or disease-specific survival. This categorization, added to patient clinical data, may facilitate the choice of therapy, minimizing treatment failure and increasing disease control.

## Introduction

Head and neck cancer (HNC) is a significant cause of mortality and morbidity worldwide, presenting approximately 600,000 new cases yearly [1], whereas tumors of the oral cavity contribute with 389,000 new cases per year, with a mortality rate of 50% [2].

Currently, the most important HNC prognostic factor is the presence of regional lymph node metastases, which correlates with a 50% reduction in life expectancy [2]–[4], however, micrometastases may not be detected by routine histology [5].

Several factors are responsible for the modulation of tumoral growth and patient prognosis. Throughout the years, factors that alter proliferation and apoptosis have received a lot of attention. It is believed that disequilibrium between proliferation and apoptosis may be the key factor in tumor development and prognosis [6].

Programmed cell death plays a critical role in the development and homeostasis of multicelullar organisms [6]. This complex process involves several genes, as well as mutations and polymorphisms that may lead to deficient death signaling and potentiation of tumor aggressiveness. Some tumor cells have acquired the ability to overcome apoptosis or to induce apoptosis of tumor-specific lymphocytes, favoring tumor progression [7]. Apoptosis resistance is a capacity shared by most malignancies. Subversion of apoptotic pathways is a major mechanism in cancer devopment, being also related with tumor aggressiveness, histological differentiation and prognosis [8], [9].

FAS (CD95), a member of the TNF family, is a transmembrane protein with cystein rich extracellular domains and a death cytoplasmatic domain, common to all family members and essential in the translation of the death stimulus [10], [11]. Immediately after the receptor stimulation by the FASL ligand (CD95L), the apoptotic signal is transmitted through the adapter FADD (*FAS Associated Death Domain*), which converts caspase 8 zymogen into its active form, triggering the apoptosis start. Activation of this cascade will culminate into DNA fragmentation, causing radical morphological and biochemical intracellular changes [11]–[12].

FAS/FASL altered expression may cause tumor protecting immunomodulation, with a direct impact on patient prognosis [13]. In a previous study, microarray experiments compared gene expression between more aggressive oral tumors (tumors with premature metastasis; T1/T2, N+) and more benign tumors (advanced tumors without metastasis; T3/T4, N0). These results generated a list of genes with differential expression, where FAS and FASL were among the least expressed in more benign tumors, suggesting a role in tumor apoptosis resistance [14]. Owing to these results, the present study aimed to correlate FAS/FASL tumor expression with clinical variables, tumor histology and prognosis of squamous cell carcinoma of the oral cavity.

## Materials and Methods

### Ethics

This study was approved by the Research Ethics Committee of the Heliopolis Hospital on 08/12/2008 (CEP n^o^ 637) and an informed consent was obtained from all patients enrolled.

### Samples

Samples were collected by the Head and Neck Genome Project (GENCAPO), a collaborative consortium created in 2002 with more than 50 researchers from 9 institutions in São Paulo State, Brazil, whose aim is to develop clinical, genetic and epidemiological analysis of HNSCC. In this study, 60 tumoral tissue samples were obtained and used for immunohistochemical analysis of the FAS and FASL gene, within a total of 60 patients with oral squamous cell carcinomas, surgically treated at the Head and Neck Surgery Department of Heliópolis Hospital, São Paulo, Brazil, during the period of January/2002 to December/2008. The clinical follow-up was at least 48 months after surgery. Previous surgical or chemotherapic treatment, distant metastasis, no removal of cervical lymph nodes and positive surgical margins were exclusion criteria. Histopathological slides were reviewed by a senior pathologist to confirm the diagnosis and select appropriate areas for immunohistochemical analysis. Tumors were classified according to the TNM system (3^rd^ edition) [15]. Clinical, epidemiological and pathological tumor characteristics are described in Table 1 and 2.

**Table 1:** Epidemiological features.

Epidemiological features	Total	
	No.	(%)
**Gender**		
Female	8	(13.3)
Male	52	(86.7)
**Age, yr**		
Median 55, df ±10.7		
**Tobacco and Alcohol habits**		
Smoker and alcoholic	50	(83.3)
Only smoker	7	(11.7)
Only alcoholic	1	(1.7)
**Tumor sub-sities**		
Tongue	22	(36.7)
Gum	12	(20.0)
Floor mouth	21	(35.0)
Retromolar area	5	(8.3)
**Treatment**		
Only operated	60	(100.0)
Operated+irradiated	31	(51.7)
**Total**	**60**	**(100.0)**

**Table 2:** Epidemiological, clinical and pathological tumor features and their association with FAS and FASL expression.

Clinical and pathological features	Total		FAS expression			FASL expression		
			Negative	Positive	*p*	Negative	Positive	*p*
	No.	(%)	No.	No.		No.	No.	
**Stage**								
2	17	(28.3)	6	11	0.025	7	10	0.177
3	17	(28.3)	7	10		5	12	
4	26	(43.4)	19	7		15	11	
**Tumor size (T)** ¥								
T1+T2	24	(40.0)	11	13	0.233	8	16	0.297
T3	12	(20.0)	5	7		7	5	
T4	24	(40.0)	16	8		12	12	
**Lymph nodes (N)** ¥								
Absent	27	(45.0)	9	18	0.004	11	16	0.548
Present	33	(55.0)	23	10		16	17	
**Diferentiation**								
Well	26	(43.4)	16	10	0.441	18	8	0.003
Moderately	29	(48.3)	13	16		7	22	
Poorly	5	(8.3)	3	2		2	3	
**Disease specific death**								
No	32	(53.3)	11	21	<0.001	9	23	0.006
Yes	25	(41.7)	20	5		16	9	
Not available*	3	(5.0)					1	
**Disease relapse**								
No	26	(43.4)	10	16	0.080	6	20	0.007
Yes	29	(48.3)	18	11		17	12	
Not available*	5	(8.3)						
**Total**	**60**	**(100.0)**	**32**	**28**		**27**	**33**	

¥ TNM classification 3^rd^ edition.

* Not available (not considered in the statistical calculations).

### Tissue Microarray

Formalin-fixed, paraffin-embedded tissue sections of 60 primary oral squamous cell carcinomas treated at the Head and Neck Surgery Department of Heliópolis Hospital, São Paulo, Brazil, were used for immunohistochemistry (IHC) analysis. Histological characterization of all samples was done by hematoxylin and eosin staining, followed by immunohistochemistry analysis of tissue microarrays (TMA). Two 1 mm cylinders were used to represent each sample in the TMA slide (Beecher Instruments®, Silver Spring, MD, USA).

### Immunohistochemistry

**Figure 1:**
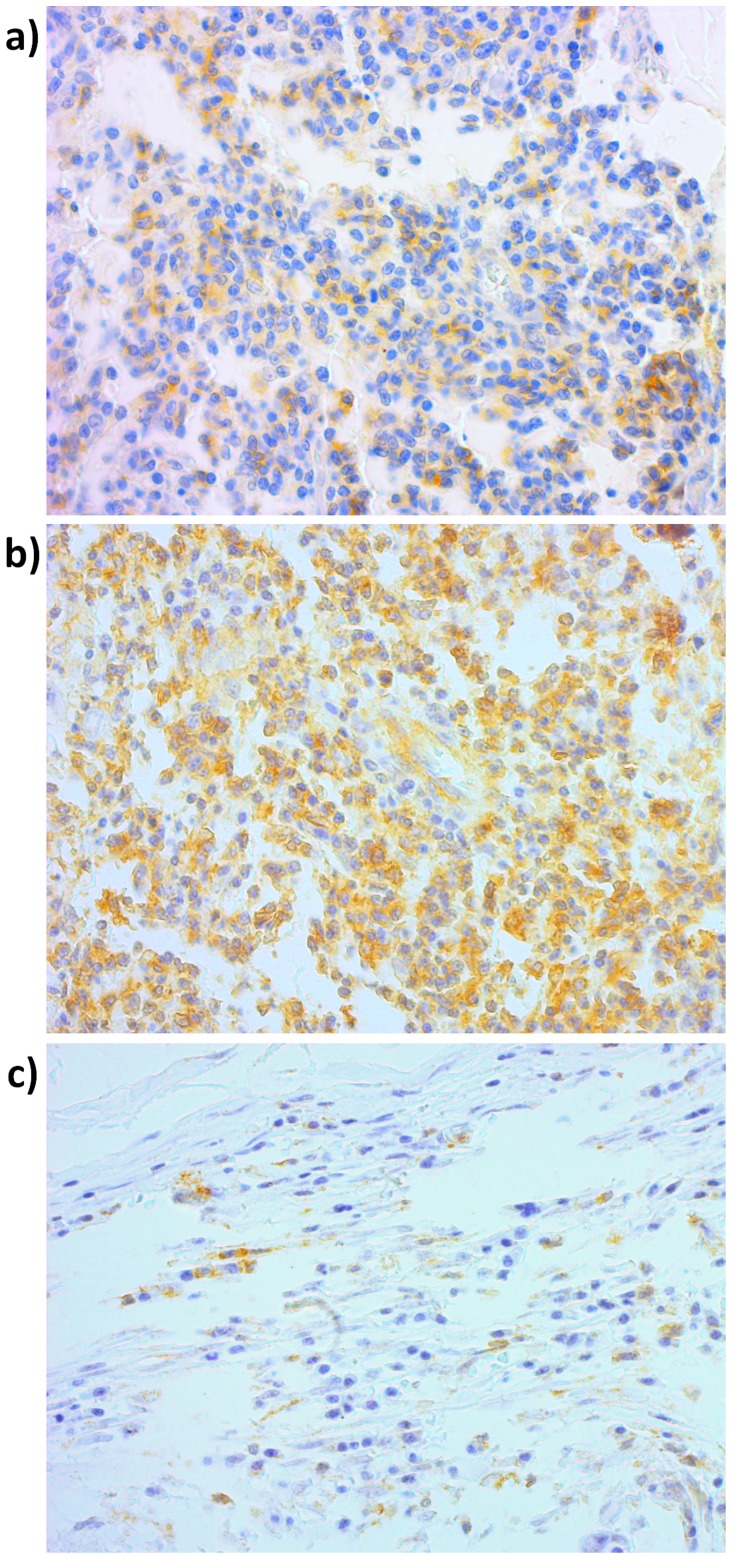
Immunohistochemical. **a.** Positive FAS expression; **b.** Positive FASL expression; **c.** Negative immunostaining. Magnification was 400×.

Anti-FAS monoclonal antibody and anti-FASL monoclonal antibody (Santa Cruz Biotechnology®, USA) were used in the IHC reaction, at a 1∶400 dilution [16]–[18]. Positive and negative controls were used. Sample scoring was performed by semi-quantitative microscopic analysis, considering the number of stained cells and signal intensity. Two spots were evaluated for each sample and a mean score was calculated. Considering the percentage of immune-positive tumor cells, a score of 1 was given when ≤10% of cells were positive; 2 when 10–50% of cells were positive and 3 when ≥50% of cells were positive. Signal intensity was scored as negative (0), weak (1), moderate (2) and strong (3). Both scores were multiplied [19], [20] and the resulting score was used to categorize FAS and FASL expression as positive (>3, Figure 1a and 1b, respectively) and negative (≤3, Figure 1c).

### Statistical Analysis

The chi square and Fisher exact tests were used for association analysis and confirmation was obtained by the Lilliefors test (significance considered when p<0.05). Multivariate logistic regression was used to obtain odds ratio (OR) and confidence intervals (CI 95%). Survival was calculated by the number of months between surgery and death for each patient or the last appointment in case the patient was alive. In order to calculate disease-free survival, the time endpoint was the date of disease relapse. The Kaplan-Meier model was used for survival analysis, using the Wilcoxon p-value and the Cox Proportional Hazards to adjust p-values and obtain hazard ratio (HR). Statistical calculations were performed using the Epi Info® v3.4.3, 2007 and Statsoft Statistica® v7.0.61.0 softwares.

## Results

### FAS Expression

FAS expression was studied in 60 tumors, of which 28 were positive (46.7%) and 32 were negative (53.3%). FAS expression did not show a significant association with tumor characteristics such as size (p = 0.233) and differentiation grade (p = 0.441), but was significantly associated with positive lymph nodes (p = 0.004, Table 2). Multivariate analysis showed that negative FAS expression was an independent marker for lymph node positivity (OR = 5.02, CI = 1.34–18.75, Table 3).

**Table 3:** Multivariate analysis of the relationship between clinical, pathological tumor features and survival with FAS and FASL expression.

Variables	Logistic regression						Cox proportional hazard			
	Lymph-nodes		Disease relapse		Disease specific death		Disease-free survival		Disease-specific survival	
	OR (95% CI)¥	*p* ¥	OR (95% CI)¥	*p* ¥	OR (95% CI)¥	*p* ¥	HR (95% CI)§	*p* §	HR (95% CI)§	*p* §
										
*FAS expression*										
Positive	1		1		1		1		1	
Negative	5.02 (1.34–18.75)	0.017	1.49 (0.39–5.78)	0.561	4.59 (1.01–21.51)	0.050	1.66 (0.69–3.97)	0.257	3.73 (1.16–11.95)	0.027
*FASL expression*										
Positive	1		1		1		1		1	
Negative	1.22 (0.30–5.00)	0.780	5.51 (1.32–23.04)	0.019	6.06 (1.05–35.06)	0.044	2.58 (1.03–6.46)	0.044	2.14 (0.73–6.30)	0.166
*Tumor size (T)*										
T1+T2	1		1		1		1		1	
T3	1.62 (0.30–8.67)	0.576	1.63 (0.29–9.25)	0.581	2.32 (0.33–16.20)	0.395	2.31 (0.73–7.35)	0.156	3.00 (0.76–11.91)	0.118
T4	4.44 (1.08–18.20)	0.038	2.68 (0.62–11.55)	0.186	2.76 (0.51–14.84)	0.236	2.05 (0.77–5.50)	0.152	1.97 (0.63–6.22)	0.245
*Differentiation*										
Well	1		1		1		1		1	
Moderately	3.56 (0.81–15.63)	0.092	1.09 (0.24–4.96)	0.909	1.66 (0.26–10.44)	0.589	1.57 (0.57–4.35)	0.385	1.84 (0.56–6.05)	0.318
Poorly	6.07 (0.45–81.73)	0.174	0.28 (0.03–2.97)	0.291	7.19 (0.37–139.86)	0.193	0.54 (0.11–2.79)	0.465	1.94 (0.41–9.17)	0.405
*Lymph-nodes*										
Absent	–	–	1		1		1		1	
Present	–	–	4.07 (0.48–34.40)	0.197	13.55 (0.94–195.73)	0.056	2.28 (0.62–8.33)	0.214	3.49 (0.78–15.65)	0.102
*Irradiated*										
No	–	–	1		1		1		1	
Yes	–	–	0.17 (0.02–1.27)	0.085	0.30 (0.02–3.68)	0.344	0.30 (0.09–0.97)	0.044	0.52 (0.17–1.56)	0.241

OR – Odds ratio; HR – Hazard ratio; CI – Confidence interval.

¥ Values adjusted by multivariate logistic regression.

§ Values adjusted by Cox proportional hazards.

FAS expression was significantly associated with disease specific death (p<0.001, Table 2) and multivariate analysis showed that negative expression was an independent death risk factor, increasing risk 4 times when compared to positive expression (OR = 4.59, CI = 1.01–21.51, Table 3). Nonetheless, FAS expression was not correlated with disease relapse (p = 0.080, Table 2).

**Figure 2:**
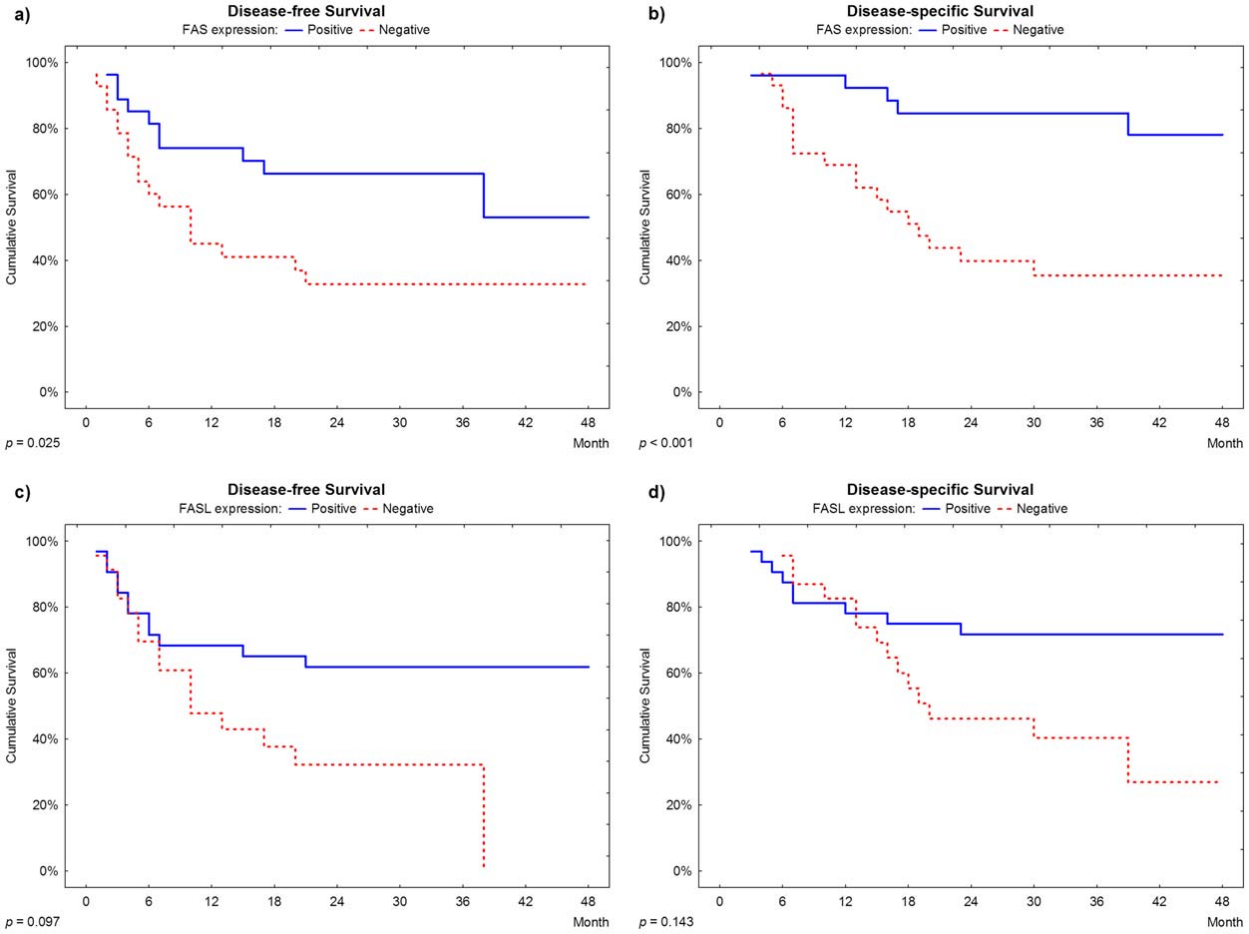
Survival plots. **a. and b.:** Disease-free survival and disease-specific survival according to FAS expression; **c. and d.:** Disease-free survival and disease-specific survival according to FASL expression.

Disease-free and disease-specific survival were significantly correlated with FAS expression (p = 0.025 and p<0.001, respectively). According to a 24 month after surgery follow up, approximately 70% of cases with negative expression presented disease relapse, as compared to approximately 25% of recurrence in patients with positive expression of FAS (Figure 2a). Additionally, according to a 36 month after surgery follow up, approximately 65% of cases with negative expression died of disease specific causes, as compared to 15% of deaths in patients with positive expression of FAS (Figure 2b). Multivariate analysis revealed that a negative expression of FAS was an independent marker for earlier disease specific death, showing a 3-fold increased risk when compared to positive expression (HR = 3.73, CI = 1.16–11.95, Table 3), but the same association was not found for disease relapse (HR = 1.66, CI = 0.69–3.97, Table 3).

### FASL Expression

Regarding FASL, 33 (55.0%) tumors showed positive expression, whereas 27 (45.0%) were negative. FASL expression was significantly associated with differentiation grade (p = 0.003), but was not associated with tumor size (p = 0.297) or positive lymph nodes (p = 0.548, Table 2).

FASL expression did significantly correlate with disease relapse (p = 0.007) and disease specific death (p = 0.006, Table 2). Multivariate analysis showed that negative FASL was an independent marker of disease relapse and disease specific death, representing an increased risk of over 6 times for each, when compared to a positive expression (respectively, OR = 5.51, CI = 1.32–23.04 and OR = 6.06, CI = 1.05–35.06; Table 3).

In contrast, disease-free and disease-specific survival were not associated with FASL expression (p = 0.143 and p = 0.097, respectively, Figure 2c and 2d).

### FAS/FASL Profiles

In an attempt to combine FAS and FASL expression results, we categorized the FAS/FASL profile in three classes: low risk (positive FAS and FASL expression); intermediate risk (negative expression of one marker) and high risk (negative expression of both markers). Frequencies of each FAS/FASL profiles were 20 (33.3%), 21 (35.0%) and 19 (31.7%), respectively for low, intermediate and high risk.

**Figure 3:**
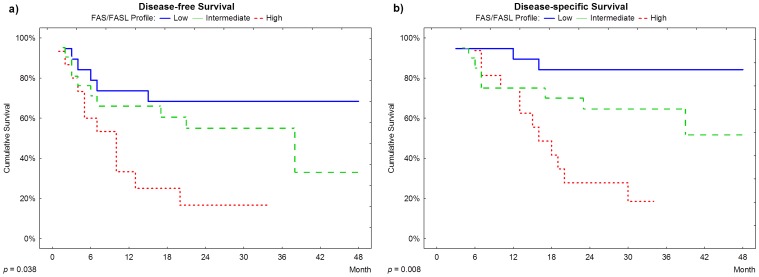
Survival plots. **a. and b.:** Disease-free survival and disease-specific survival according to FAS/FASL profile.

Disease-free and disease-specific survival were significantly correlated with FAS/FASL profiles (p = 0.038 and p = 0.008, respectively). On a 24 month after surgery follow up, 80% of cases classified as high risk had relapsed and approximately 70% died of disease-specific causes, compared to approximately 30% of relapse and 15% of death in patients classified as low risk (Figure 3a and 3b). Multivariate analysis revealed that the high risk category is an independent marker for earlier disease relapse and disease-specific death, with approximately 4- and 6-fold increased risk, respectively, when compared to the low risk profile (HR = 3.80, CI = 1.19–12.52 and HR = 6.43, CI = 1.45–28.55).

## Discussion and Conclusions

Apoptosis is a physiological process of cell number control, which plays an important role in cellular homeostasis and embryonic development [21]–[24]. Cell population is defined by a balance between proliferation and survival and disruption of this balance can lead to cancer growth [25]–[32].

The extrinsic apoptosis pathway can be triggered by enzymes of the TNF family, including FAS and FASL. FASL positive T-cells can eliminate FAS positive tumoral cells by inducing apoptosis [10], [12]. Therefore, reduction or loss of FAS expression may result in decreased sensitivity of tumoral cells to cytotoxic activity, impairing apoptosis.

FAS expression has been previously associated with tumor apoptosis in the stomach [13], esophagus [33] and liver [34]. In addition, FAS/FASL diminished expression correlates with worse prognosis in lung [35] esophageal [36], larynx [37], colorectal [38] and gastric [39] tumors.

In agreement with the literature, our results show that negative FAS expression correlates with lymph node metastasis (5 times increased risk). When compared with positive expression, negative expression was significantly associated with cancer related deaths and shorter disease-free and disease-specific survival. Multivariate analysis confirmed that negative FAS expression was an independent risk factor for death and disease-specific survival reduction, increasing risk approximately 5 times for each. Our results also showed that negative FASL expression was associated with increased disease relapse and disease-related deaths. Multivariate analysis confirmed that FASL negative expression was an independent risk factor for disease relapse and death, increasing risk up to 6 times when compared to positive expression. However, FASL expression was not related to worse disease-free survival or disease-specific survival.

In contrast with our results, other studies have revealed higher FASL expression as a marker of worse prognosis in esophageal [36] and lung [40]–[42] tumors. Their hypothesis relies on tumor FASL expression as a T-cell apoptosis inducer, resulting in lower tumor attack by the immune system [43]–[45]. However, our results support the hypothesis that the immune system response is already compromised in oral cancer, most likely because it is a tobacco/alcohol associated disease [46]. As previously reported, chronic alcohol consumption impairs Natural Killer cell (NK) activity and decreases NK cell number, therefore affecting their ability to destroy tumor cells. [47]. In addition, several studies have reported a similar decrease in number and activity of NK cells in smokers [48]; [49], in which cases a lower production of interferon-c and TNF-a cytokines is observed [50]. Based on these facts, our hypothesis predicts that the oral immune response is attenuated in patients with chronic tobacco and alcohol consumption, therefore in these individuals, a lack of FASL may represent a loss of the extrinsic apoptosis signal in tumor cells, conferring a worse prognosis.

In summary, our results correlate a negative FAS/FASL expression with worse prognosis in oral squamous cell carcinoma patients, suggesting that these proteins play important roles in oral cancer cell apoptosis.
